# PDBe and PDBe‐KB: Providing high‐quality, up‐to‐date and integrated resources of macromolecular structures to support basic and applied research and education

**DOI:** 10.1002/pro.4439

**Published:** 2022-09-28

**Authors:** Mihaly Varadi, Stephen Anyango, Sri Devan Appasamy, David Armstrong, Marcus Bage, John Berrisford, Preeti Choudhary, Damian Bertoni, Mandar Deshpande, Grisell Diaz Leines, Joseph Ellaway, Genevieve Evans, Romana Gaborova, Deepti Gupta, Aleksandras Gutmanas, Deborah Harrus, Gerard J. Kleywegt, Weslley Morellato Bueno, Nurul Nadzirin, Sreenath Nair, Lukas Pravda, Marcelo Querino Lima Afonso, David Sehnal, Ahsan Tanweer, James Tolchard, Charlotte Abrams, Roisin Dunlop, Sameer Velankar

**Affiliations:** ^1^ European Molecular Biology Laboratory European Bioinformatics Institute Hinxton; ^2^ CEITEC – Central European Institute of Technology Masaryk University Brno Czech Republic; ^3^ National Centre for Biomolecular Research, Faculty of Science Masaryk University Brno Czech Republic

**Keywords:** bioinformatics, databases, protein data bank, structural biology

## Abstract

The archiving and dissemination of protein and nucleic acid structures as well as their structural, functional and biophysical annotations is an essential task that enables the broader scientific community to conduct impactful research in multiple fields of the life sciences. The Protein Data Bank in Europe (PDBe; pdbe.org) team develops and maintains several databases and web services to address this fundamental need. From data archiving as a member of the Worldwide PDB consortium (wwPDB; wwpdb.org), to the PDBe Knowledge Base (PDBe‐KB; pdbekb.org), we provide data, data‐access mechanisms, and visualizations that facilitate basic and applied research and education across the life sciences. Here, we provide an overview of the structural data and annotations that we integrate and make freely available. We describe the web services and data visualization tools we offer, and provide information on how to effectively use or even further develop them. Finally, we discuss the direction of our data services, and how we aim to tackle new challenges that arise from the recent, unprecedented advances in the field of structure determination and protein structure modeling.

## 1 INTRODUCTION

The Worldwide Protein Data Bank (wwPDB) is the consortium responsible for operating the PDB archive.^1^ Its members are the Protein Data Bank in Europe (PDBe; pdbe.org),^2^ RCSB Protein Data Bank,^3^ Protein Data Bank Japan (PDBj),^4^ the Biological Magnetic Resonance Bank (BMRB),^5^ and the Electron Microscopy Data Bank (EMDB).^6^ The archiving of experimentally determined macromolecule structures, painstakingly determined by researchers of the global scientific community, is a fundamental and valuable task.^1^ It is estimated that reproducing the experimentally determined protein and nucleic acid structures currently in the PDB archive would cost around 19 billion dollars.^7^ The wwPDB members collaborate on data archiving, but in addition each site provides its own data‐access services, software, and enriched data sets.

Open access to the wealth of macromolecular structure data in the PDB archive enables research and software development in several scientific fields. Structure‐based drug discovery, structural bioinformatics studies, experimental determination of new protein structures and scientific software development all benefit greatly from the data stored in the PDB archive. Indeed, the recent spectacular advances in protein structure prediction using Artificial Intelligence (AI) approaches, as demonstrated by AlphaFold^8^ and RoseTTaFold,^9^ would not have been possible without the training set of experimentally determined structures in the PDB archive.

While the three‐dimensional coordinates of macromolecules are essential for answering a variety of scientific questions, these data are often insufficient if they lack a biological context.^10^, ^11^ A lot of data resources and scientific software specialize in providing parts of this biological context through functional, biophysical, and biochemical annotations.^12^, ^13^, ^14^, ^15^ Yet, a significant barrier to taking systematic advantage of these valuable annotations is that they are often lacking in FAIRness,^16^ that is, they might not be sufficiently findable, accessible, interoperable or reusable. In particular, findability is hampered by the fragmented nature of these data resources, making it difficult for researchers to keep track of the location and existence of the annotations. Interoperability is also a significant challenge, as each data provider may use custom data formats, ontologies, and definitions.

PDBe has worked with research groups and scientific service teams to establish the PDBe Knowledge Base (PDBe‐KB; pdbekb.org) consortium to tackle these challenges.^11^ PDBe‐KB is an open, collaborative consortium that provides FAIR access to the biological context of macromolecular structure data. It is one of the flagships of the ELIXIR 3D‐BioInfo Community, a group of researchers and software developers working on improving all aspects of data management in the field of structural bioinformatics.^17^ PDBe‐KB has expanded significantly since its inception in 2018, and provided valuable data sets and tools for the scientific community.

In recent years, both PDBe and PDBe‐KB have grown in terms of the amount of data and the number of services they provide. The PDB archive is growing steadily, with high‐resolution structures determined using electron cryo‐microscopy (cryo‐EM) rising swiftly. The number of Cryo‐EM depositions has now overtaken those of nuclear magnetic resonance (NMR) methods, and cryo‐EM depositions are approaching those of X‐ray crystallography, historically the most prevalent experimental method used to determine the structures in the PDB.

While the core PDB data is the same for every wwPDB consortium member, PDBe provides additional unique services. These have been driving an increase in usage in recent years, with almost half a million monthly unique users visiting PDBe and PDBe‐KB entry pages and growing access of PDBe data through its public application programming interface (API).^18^ The amount of data in PDBe‐KB has also increased sharply, following integration of a growing number of structural, functional, biophysical, and biochemical annotations contributed by the PDBe‐KB partner resources. These data allow us to provide an increasingly comprehensive context for PDB structures, as demonstrated by the so‐called “aggregated views” pages of PDBe‐KB, which collate all available structural data and their annotations for a specific protein of interest.^19^

Here, we give a detailed overview of the PDBe and its sister resource, the PDBe‐KB.^2^, ^11^ We will discuss the infrastructure of these massive databases and provide descriptions of the services, data sets and data visualization tools developed under the umbrella of PDBe and PDBe‐KB. We will subsequently highlight the availability of high‐quality, up‐to‐date training materials and conclude by presenting an outlook for the future of these data resources.

## 2 INFRASTRUCTURE

The PDBe team develops and maintains several public and open‐access data resources and scientific tools. Some of these are under the umbrella of PDBe, while others are related to the PDBe‐KB, the AlphaFold Protein Structure Database,^20^ or the 3D‐Beacons Network (Table 1). The following sections provide a detailed overview of these services.

**TABLE 1:** Services of the PDBe team

Name of service	Brief description	(Example) URLs
3D‐Beacons API	Programmatic access to models	https://3d‐beacons.org/api
3D‐Beacons network	Access to experimental and theoretical structures	https://3d‐beacons.org
AFDB entry pages^20^	View single AlphaFold predictions	https://www.alphafold.ebi.ac.uk/entry/Q5VSL9
Aggregated views of proteins^11^	View aggregated structural data for proteins	https://pdbe‐kb.org/proteins/Q14676
Density server	Access to volumetric density data	https://www.ebi.ac.uk/pdbe/densities/doc.html
FunPDBe annotations^19^	JSON files containing functional annotations	http://ftp.ebi.ac.uk/pub/databases/pdbe‐kb/annotations/
Model server	Access to (sub‐)structure models of molecules	https://molstar.org/docs/data‐access‐tools/model‐server/
Neo4j database	Downloadable graph database of PDBe and PDBe‐KB data	https://www.ebi.ac.uk/pdbe/pdbe‐kb/graph‐download
PDBe API^18^	Programmatic access to PDBe and PDBe‐KB data	https://www.ebi.ac.uk/pdbe/pdbe‐rest‐api
PDBe component library	Reusable web components from PDBe	https://www.ebi.ac.uk/pdbe/pdb‐component‐library/
PDBe download service	Download PDB data for lists of PDB entries	https://www.ebi.ac.uk/pdbe/download/docs
PDBe entry pages^2^	View single PDB entries	https://pdbe.org/3bow
PDBe Mol*^21^	Interactive 3D molecular viewer	https://github.com/molstar/pdbe‐molstar
PDBe PISA	Analyse molecular assemblies	https://www.ebi.ac.uk/msd‐srv/prot_int/pistart.html
PDBe ProtVista^22^	Interactive 2D sequence feature viewer	https://github.com/PDBeurope/protvista‐pdb
PDBe‐KB component library	Reusable web components from PDBe‐KB	https://github.com/PDBe‐KB?q=component
PDBeChem	Search system for small molecules	https://www.ebi.ac.uk/pdbe‐srv/pdbechem/
PDBeFold	Structure‐based search	https://www.ebi.ac.uk/msd‐srv/ssm/
SIFTS^23^	Mapping of PDB entries to UniProt and other databases	https://www.ebi.ac.uk/pdbe/docs/sifts/

**FIGURE 1:**
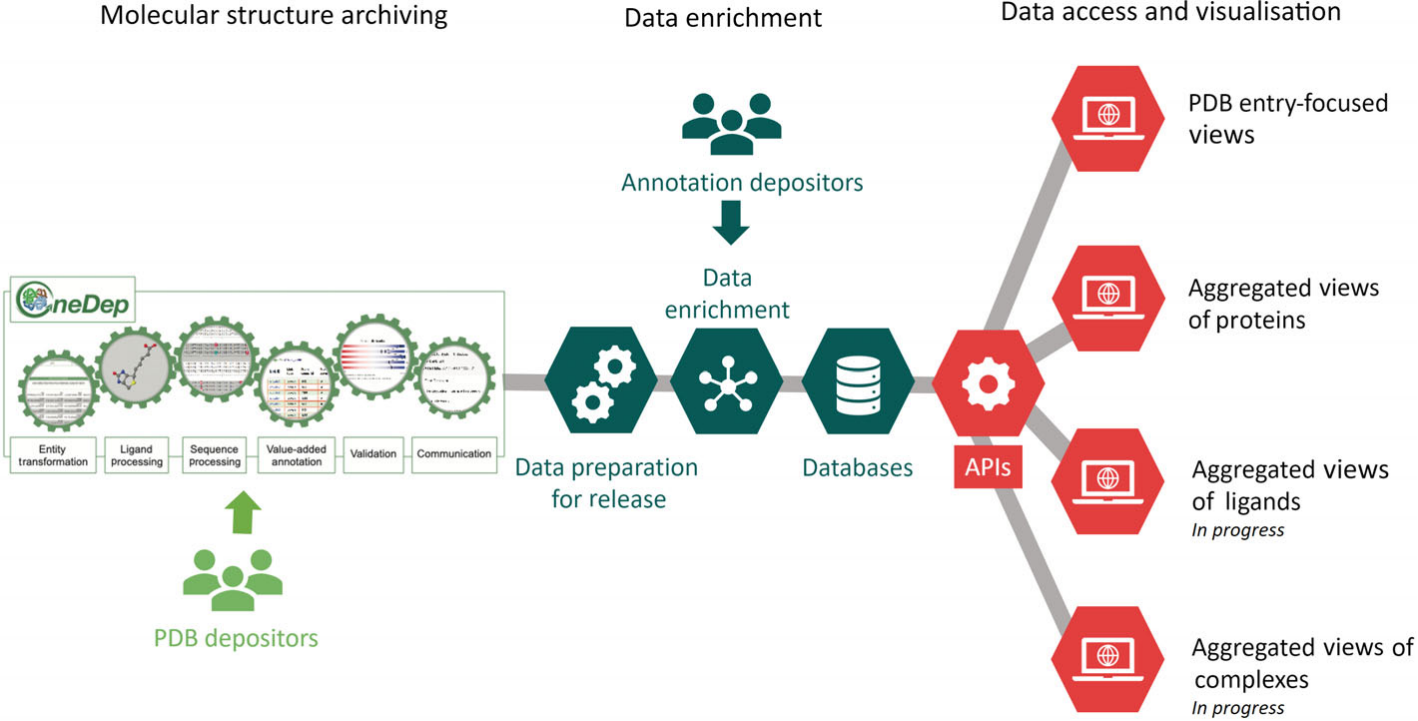
Overview of the infrastructure developed by the PDBe team The main wwPDB deposition system (OneDep) captures the core PDB data from depositors worldwide (light green). We enrich the core PDB data with annotations provided by PDBe‐KB partner databases and archive the data in internal databases (dark green). Finally, we provide data‐access mechanisms and visualizations for every aspect of the structural data we manage (red). PDBe, Protein Data Bank in Europe; PDBe‐KB, PDBe Knowledge Base; wwPDB, Worldwide Protein Data Bank

The infrastructure can be divided into several well‐defined, and stand‐alone components: data‐deposition systems, databases, programmatic access software, and data visualization (Figure 1). The wwPDB common data‐deposition system, OneDep,^24^ handles the processing and curation of new PDB entries, while a separate system processes and integrates functional and biophysical annotations from PDBe‐KB partners. All the data are loaded into our internal databases, and we provide programmatic access to data both for external users and to PDBe services. The PDBe entry pages and the PDBe‐KB aggregated views of proteins display the core PDB data, and enriched structural and functional annotations from PDBe‐KB partners and our internal data pipelines.

### 2.1 Deposition systems

PDBe is a core member of the wwPDB consortium.^1^ All wwPDB members use OneDep, the common deposition, annotation, and validation system to support the deposition of macromolecular structure data and associated experimental data to the PDB and EMDB core archives. The OneDep system is collaboratively developed by PDBe, RCSB PDB, PDBj, and EMDB.^2^, ^3^, ^4^, ^6^

Whereas the OneDep system serves the depositors of new PDB and EMDB entries, the PDBe‐KB deposition system serves data resources that are members of the PDBe‐KB consortium. This deposition system focuses on functional and biophysical annotations that can enrich core PDB data. The basis of the deposition system is a data‐exchange format collaboratively developed by the PDBe‐KB consortium and maintained by the PDBe team. This format is a JSON (JavaScript Object Notation) specification and aims to capture the minimal required metadata to describe residue‐level annotations. For example, the schema supports archiving information on which PDB residues are predicted to form a druggable pocket or which residues have known variants that may have a deleterious effect on the stability of the protein chain.^12^, ^25^, ^26^ The data‐exchange format schema is available at https://github.com/PDBe-KB/funpdbe-schema.

Every PDBe‐KB consortium member converts their specific annotations to the agreed data‐exchange format and transfers these JSON file sets to the PDBe team. Each week, we process, validate and merge the annotations from the JSON files with the core PDB data. We also make the JSON files publicly available at http://ftp.ebi.ac.uk/pub/databases/pdbe-kb/annotations/. These integrated annotations provide the biological context of proteins to users through our services, for example the aggregated views of proteins (e.g., https://www.ebi.ac.uk/pdbe/pdbe-kb/proteins/P0DTD1). As of July 2022, the PDBe‐KB partners have provided over 80 million residue‐level annotations for over 185,000 PDB entries. These annotations provide useful information about predicted and observed ligand‐binding sites, potential druggable pockets, predicted post‐translational modifications (PTMs), catalytic sites, backbone flexibility, interface classification, and topology classifications, to name a few.^11^

### 2.2 Databases

**FIGURE 2:**
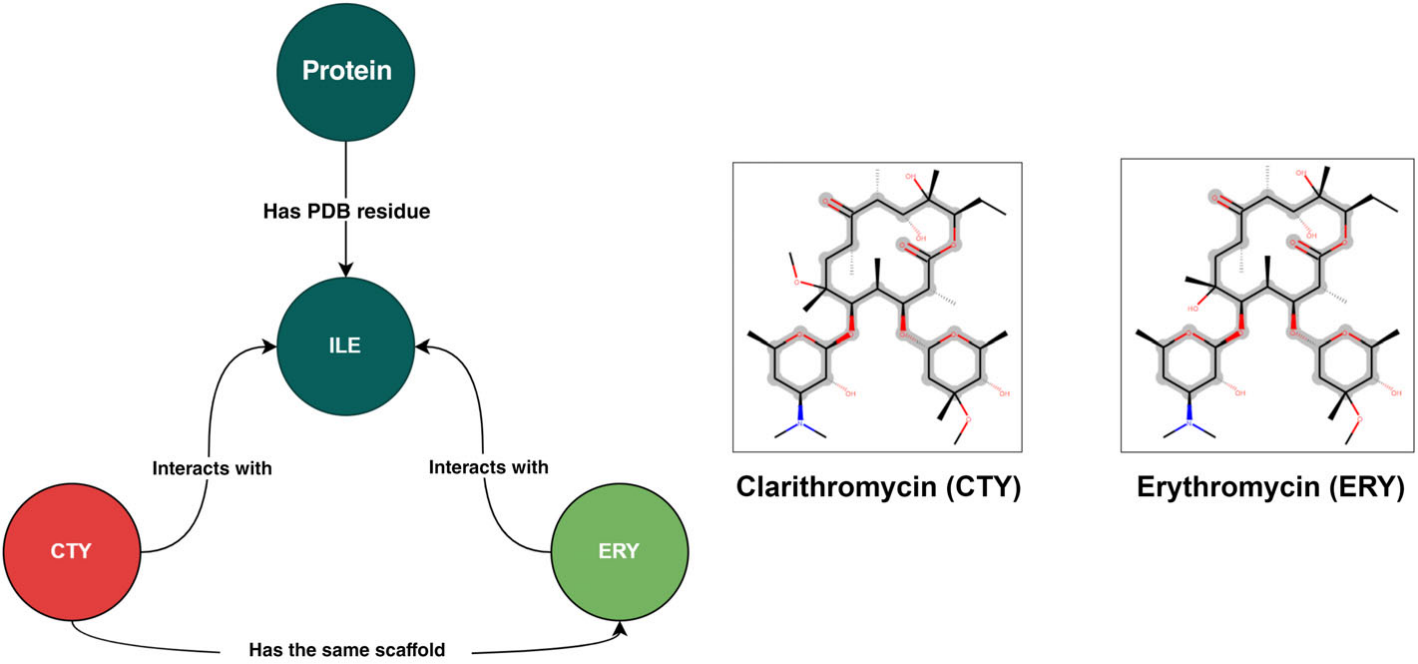
Example of using the PDBe graph database. The PDBe graph database is a powerful tool for research and discovery. In the example above, we queried the graph to identify ligand molecules in the PDB archive that bind to the same binding site and have the same molecular scaffolds. One of the ligand pairs matching this query is clarithromycin and erythromycin, a pair of antibiotics. Both molecules interact with the same amino acids of aminoglycoside phosphotransferase, for example with residue ILE105. These interactions can be viewed at https://www.ebi.ac.uk/pdbe/pdbe‐kb/proteins/Q47396/ligands. PDBe, Protein Data Bank in Europe

We integrate the PDBe‐KB annotations with the core PDB data using two approaches to data archival: a Neo4j graph database, that allows us to aggregate and perform sophisticated analysis and querying, and an Oracle relational database, which is more performant in case of simpler queries. The Oracle database currently powers the majority of the PDBe entry pages and PDBe search services. The Neo4j graph database powers the PDBe‐KB aggregated views pages. Additionally, the graph database is a powerful tool that researchers can query using complex sub‐graph patterns to answer specific scientific questions. For example, the graph database can easily help identify ligand molecules in the PDB that have the same Murcko scaffold and bind to the same binding site of a target protein (Figure 2). See Supplementary Material SS1 for the relevant Cipher query.

We make a weekly updated copy of the graph database available to the scientific community at https://www.ebi.ac.uk/pdbe/pdbe-kb/graph-download. Installing a local copy of this database allows researchers to use it as an in‐house discovery tool. It is especially powerful if users integrate their own data into the graph, allowing them to map their data to that in the PDB seamlessly. This can be done by downloading and running a copy of the PDBe graph database, and modifying and loading new nodes and edges. We provide detailed documentation of the schema of the graph database at https://www.ebi.ac.uk/pdbe/pdbe-kb/schema.

### 2.3 Programmatic access

We provide programmatic access to data from both the relational and the graph database through a rich set of API endpoints. More than 90 endpoints provide data in JSON format that addresses specific use cases, such as mappings between PDB chains and UniProt accessions or lists all observed ligand‐binding sites in a PDB structure.^18^ To create these mappings, we use data from the Structure Integration with Function, Taxonomy, and Sequence (SIFTS)^23^ resource. In addition to mappings between PDB residues and UniProt residues, SIFTS also provides annotations from the IntEnz, GO, InterPro, Pfam, CATH, SCOP, PubMed, Ensembl, and Homologene resources.^27^, ^28^, ^29^, ^30^, ^31^, ^32^

Comprehensive documentation of both SIFTS‐related and other API endpoints is available at https://www.ebi.ac.uk/pdbe/pdbe-rest-api. Users can search and test all API endpoints on these documentation pages, helping them find those relevant to their specific scientific analysis or bioinformatics pipeline needs.^18^ The API endpoints are also used by the PDBe services and by external data resources and scientific software. For example, the API endpoints exposing data from the PDBe graph database power the PDBe‐KB aggregated views of proteins and provide annotations to the 2D sequence‐feature viewer, PDB ProtVista,^22^ on the PDBe entry pages. Other data resources, such as the AlphaFold Protein Structure Database, UniProt, and GeneCards, depend on data accessed through our API endpoints.^20^, ^33^, ^34^

## 3 PROTEIN DATA BANK IN EUROPE WEB SERVICES

PDBe offers web pages dedicated to describing individual PDB structures; for example, https://pdbe.org/3bow provides all the information for PDB entry 3bow. These entry pages offer direct access to files for download, display most of the textual metadata (such as biological function), experimental conditions, and validation information, and include interactive data‐visualization tools to help users understand the molecular structure data and their annotations.

**FIGURE 3:**
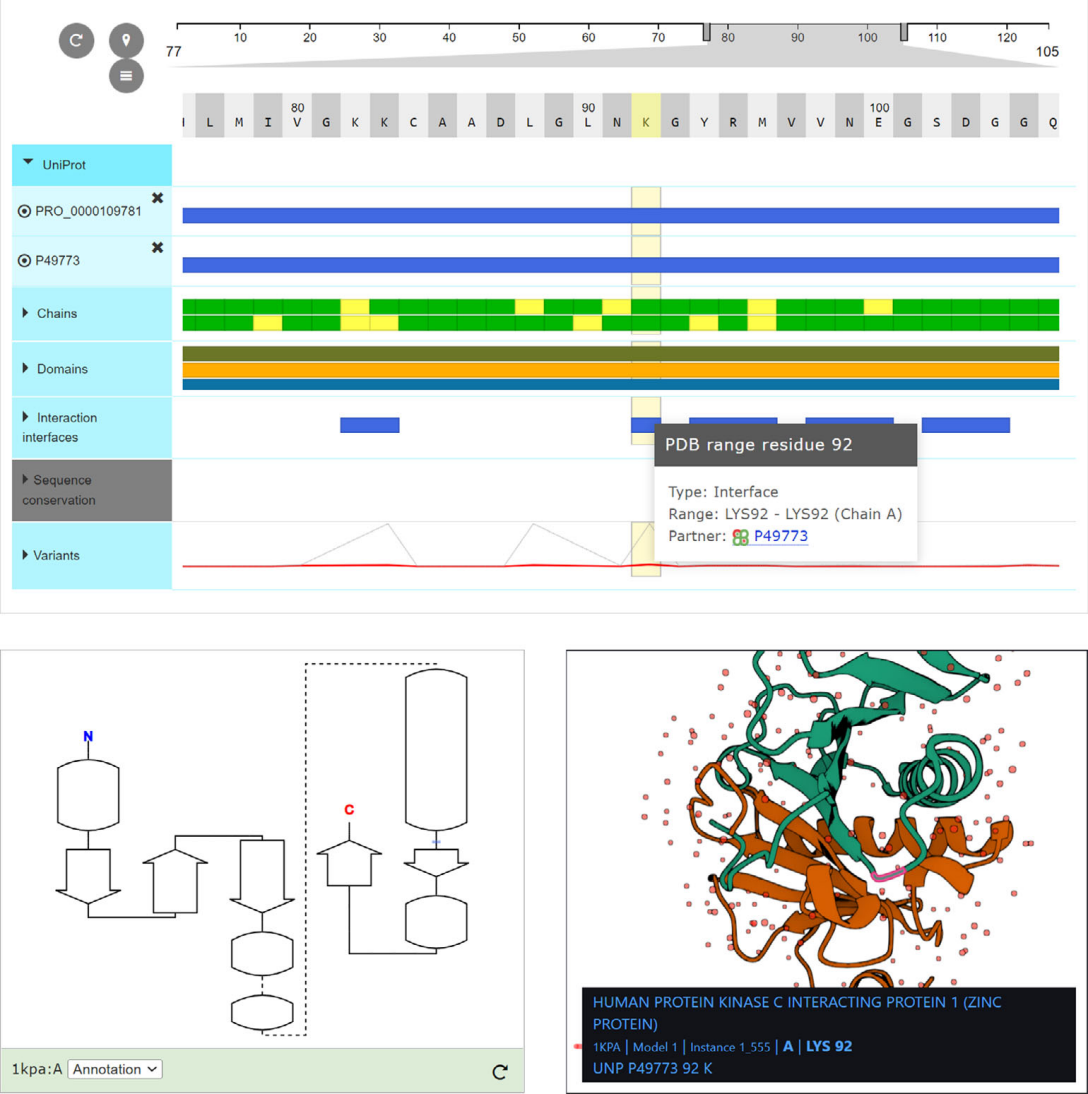
PDBe Mol*, PDBe ProtVista and the protein topology viewer The three main data‐visualization tools as displayed on the PDBe entry page of PDB 1KPA at https://www.ebi.ac.uk/pdbe/entry/pdb/1kpa/protein/1, corresponding to the human HINT1 protein. PDB ProtVista is at the top, the 2D topology viewer is at the bottom left, and PDBe Mol* is at the bottom right. Using these data‐visualization tools, users can do visual analyses, for example, examining the potential effect of a known mutation at LYS92. It becomes apparent that this residue is in a loop region that is part of the dimeric interaction interface. Therefore, one can speculate that a mutation might disrupt or destabilize the dimerization of HINT1. PDBe, Protein Data Bank in Europe

There are three primary, interactive data‐visualization tools on the PDBe entry pages: the 3D molecular graphics viewer, PDBe Mol*,^21^ the 2D sequence‐feature viewer, PDB ProtVista,^22^ and the 2D topology viewer for proteins and RNA molecules (Figure 3).

PDBe Mol* is a PDBe‐specific implementation of the Mol* suite.^21^ Its source code is available at https://github.com/molstar/pdbe-molstar. PDBe‐Mol* includes only those functionalities of the Mol* suite, that are essential for the data visualizations on PDBe, PDBe‐KB, and AlphaFold DB pages.^2^, ^11^, ^20^ This interactive molecular graphics viewer can load, display, and save 3D visualizations of macromolecules, provide different rendering styles and support superposition views to allow users to compare various PDB structures of the same protein. PDBe Mol* is designed to have intuitive controls to ease basic usage, and documentation, with detailed interactive demos and tutorials on how to embed Mol* are provided at https://github.com/molstar/pdbe-molstar/wiki.

PDB ProtVista is another primary data‐visualization tool we use on PDBe and PDBe‐KB pages.^22^ It is a 2D sequence‐feature viewer that allows us to display the functional and biophysical annotations provided by the PDBe‐KB partner resources. We make the source code of PDBe ProtVista available at https://github.com/PDBeurope/protvista-pdb and provide detailed demos and guides on integrating it with other data resources at https://github.com/PDBeurope/protvista-pdb/wiki. This viewer displays annotations in so‐called “tracks.” In its current implementation, PDB ProtVista has four types of tracks: a segment‐based track, a site/residue‐based track, a variants track, and a sequence‐conservation track (Supplementary Material SS2).

Finally, the topology viewer includes two distinct web components that display protein topology and RNA topology in a consistent style. Protein topology data is calculated using the PDBSum software package,^35^ while for RNA topologies we generate the data using R2DT^36^ and FR3D.^37^ This 2D topology viewer communicates with PDBe Mol* and PDB ProtVista, allowing users to interactively identify and map residues between the three data visualization tools.

While Mol*, ProtVista, and the topology viewer are the main data‐visualization web components of PDBe, we have several other reusable components for specific tasks, such as providing summary information about a PDB entry or displaying residue‐interaction networks.^2^ We make all these web components available to the scientific community in our PDBe web component library at https://www.ebi.ac.uk/pdbe/pdb-component-library/.

## 4 PROTEIN DATA BANK IN EUROPE DOWNLOAD SERVICE

**FIGURE 4:**
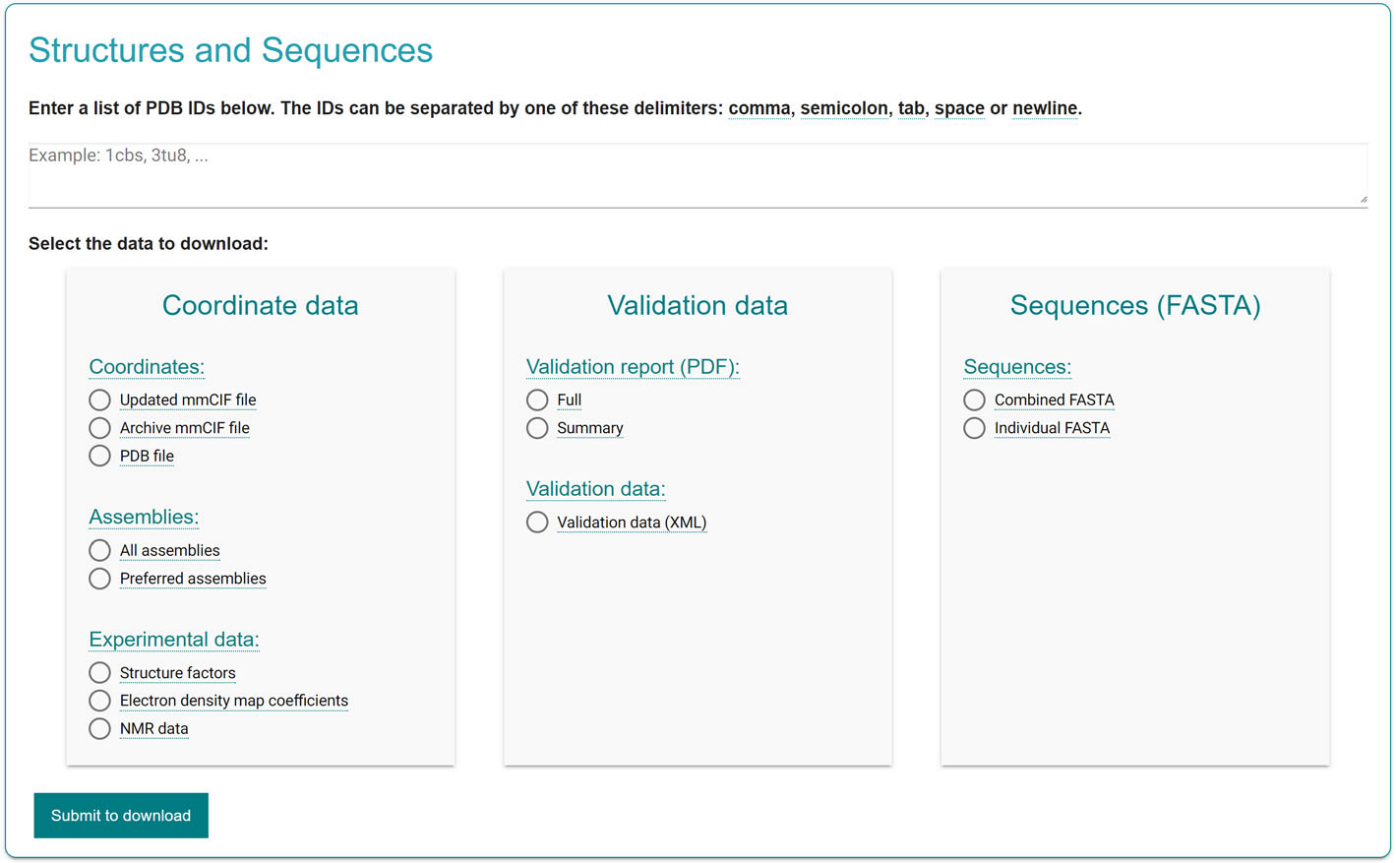
Bulk‐data download service We provide a bulk‐data download service for all aspects of PDB data. Using a list of PDB entries or small molecule identifiers (HET codes), users can retrieve large volumes of data from coordinate files to validation data and more. PDB, Protein Data Bank

Researchers are often interested in a collection of PDB entries relevant to the specific scientific question they are investigating. While downloading data for individual PDB entries is straightforward, performing bulk downloads was often more complicated than necessary. Therefore, we have created a bulk‐data download service with an intuitive user interface to help users select and download large volumes of PDB data (Figure 4). This service allows users to download coordinate data, validation data, sequences, data on ligands and other small molecules, and residue mappings between PDB and UniProt.

## 5 PROTEIN DATA BANK KNOWLEDGE BASE WEB SERVICES

**FIGURE 5:**
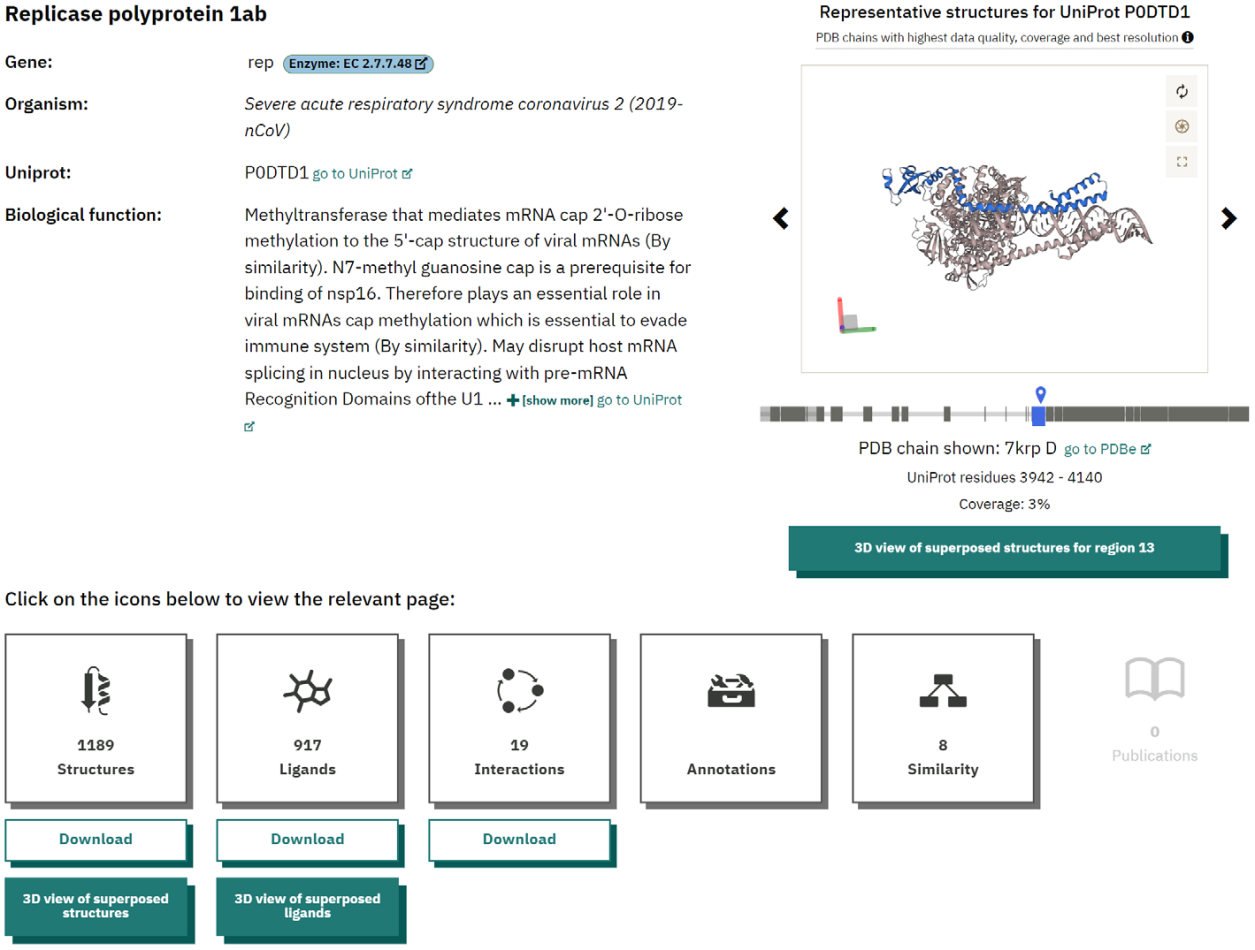
PDBe‐KB aggregated views of proteins The latest update of the PDBe‐KB aggregated views of proteins offers a streamlined user experience with better separation and description of the various data we provide access to. Each primary section, such as “structures,” “ligands,” etc. has its own subpage. This allowed us to fine‐tune the performance of the subpages while also displaying more data before. The example shown here is the Replicase polyprotein 1ab of SARS‐CoV‐2, https://pdbe‐kb.org/proteins/P0DTD1. PDB, Protein Data Bank; PDBe‐KB, PDBe Knowledge Base

Currently, the main offering of PDBe‐KB is the collection of pages that provide aggregated structurall data and functional annotations on a per‐protein basis, the so called aggregated views of proteins.^18^ We have recently redesigned these pages to provide a more intuitive user interface, while also making the pages more performant to account for the massive increase in annotations provided by PDBe‐KB partner resources (Figure 5).

The latest version of the aggregated views of proteins display functional annotations for ligands, such as whether an observed ligand is a reactant‐like (similar to product or substrate), a cofactor‐like, or a drug‐like molecule.^38^ We use internal data pipelines to create these annotations weekly, using the latest PDB data. These pipelines are available as part of our open‐source PDBe CCDutils package (https://github.com/PDBeurope/ccdutils). The pages also include biophysical descriptions such as per‐residue solvent‐accessible surface area from POPS^39^ and flexibility propensities from webNMA.^40^ Together, the annotations from the PDBe‐KB consortium place proteins in a more comprehensive biological and functional context, allowing researchers to better understand the implications of the structural data in the PDB archive.

In addition to displaying annotations and PDB structures, we also make the underlying data available to researchers and software developers. We have worked with the ELIXIR 3D‐BioInfo community to make specific benchmark datasets accessible for a number of well‐defined aspects of structural bioinformatics.^17^ Some of these datasets focus on distinct conformational states and their biological role, while others focus on training prediction methods for ligand‐binding sites, PTM sites or variants. We provide access to these datasets at http://ftp.ebi.ac.uk/pub/databases/pdbe-kb/benchmarking/.

Finally, we open‐sourced all the web components we used to build the PDBe‐KB aggregated views so that other data services can reuse these data visualizations: https://github.com/PDBe-KB?q=component.

## 6 TRAINING MATERIALS

Keeping up with the amount and types of data can at times be challenging. Therefore, it is very important that we provide up‐to‐date and user‐friendly training materials, tutorials, demos, and webinars for all our services that help users take advantage of the wealth of structure data and their functional annotations. These training materials are available from our training portal at https://www.ebi.ac.uk/pdbe/pdbe-training.

As part of the EMBL‐EBI training initiatives, we are regularly offering webinars and in‐person workshops that cover every aspect of our services from the core PDB data to programmatic access of PDBe‐KB annotations. Within the on‐demand training portfolio of EMBL‐EBI, we provide an introductory course to PDBe at https://www.ebi.ac.uk/training/online/courses/exploring-pdb-entry/. We regularly host webinars and we make the recordings available at our YouTube channel: https://www.youtube.com/user/ProteinDataBank. These webinars cover topics such as the PDBe API, the 3D molecular viewer Mol*, and how to take advantage of the PDBe‐KB aggregated views of proteins. We also have interactive tutorials covering PDBe search, API, and other PDBe tools at https://pdbeurope.github.io/api-webinars/index.html.

We follow a policy of open‐sourcing the majority of our data pipelines and data visualization tools, generally in public repositories on the GitHub platform at PDBe (https://github.com/PDBeurope) and PDBe‐KB (https://github.com/PDBe-KB), under Apache 2.0 license. The code repositories provide technical descriptions and instructions on how to install and use the tools. We provide technical documentation which includes demos, such as the ones described in previous sections related to PDBe Mol* and PDB ProtVista.

Feedback from the user community is very valuable, and drives all our work including improving the training materials, to reflect the most common use cases and address the most complicated aspects of our services.

## 7 DISCUSSION

The archival of experimentally determined structures is a fundamental service both to structural biologists and the broader scientific community. The importance of protein structure archiving was highlighted by the recent, unprecedented advances in AI‐based protein‐structure prediction, as demonstrated by AlphaFold^8^ and RoseTTaFold.^9^ Neither these nor any other prediction methods would have been possible without the painstaking effort, ingenuity, and determination of generations of structural biologists or without the open, and transparent access to protein structure data from the Protein Data Bank.^1^

The advent of these advanced computationally predicted models disrupted several fields in the life sciences, from structure‐based drug discovery and bioinformatics analysis to software development and structure determination efforts.^41^, ^42^, ^43^, ^44^, ^45^, ^46^, ^47^, ^48^ Researchers regularly use AlphaFold models to solve protein structures using their (sometimes old) experimental data.

Going forward, it becomes increasingly important to provide seamless integration of all structural data; experimental, predicted, and hybrid. Initiatives, such as the 3D‐Beacons network, are increasingly important in this aspect, allowing researchers to find and access protein structures from several different data resources.

To address the changes in the field of structural biology, we are now focusing on improving the integration between PDBe and PDBe‐KB web services, and investigating new approaches to aggregating structural data and their corresponding functional and biophysical annotations. We are exploring ways to identify and collate data around single ligand molecules observed in the PDB archive, as well as for macromolecular assemblies. The latter are often the de facto functional units in many biological systems, therefore they are at the crux of answering scientific questions even more so than individual protein structures.

The landscape of structural biology continues to change rapidly, and our services need to evolve to facilitate basic and translational research by supporting macromolecular structure depositors, specialist data resources, researchers and software developers alike.

## AUTHOR CONTRIBUTIONS

**Mihaly Varadi:** Conceptualization (equal); formal analysis (equal); investigation (equal); project administration (lead); resources (equal); software (equal); supervision (lead); visualization (supporting); writing – original draft (lead). **Stephen Anyango:** Software (equal). **Sri Devan Appasamy:** Formal analysis (equal); investigation (equal); software (equal); visualization (equal). **David Armstrong:** Data curation (equal); writing – review and editing (supporting). **Marcus Bage:** Data curation (equal); formal analysis (equal); software (equal); visualization (equal); writing – review and editing (supporting). **John Berrisford:** Conceptualization (equal); data curation (equal); project administration (equal); software (equal); supervision (equal); validation (equal); writing – review and editing (equal). **Preeti Choudhary:** Conceptualization (equal); data curation (equal); formal analysis (equal); software (equal); visualization (equal). **Damian Bertoni:** Software (equal); visualization (equal). **Mandar Deshpande:** Software (equal); visualization (equal). **Grisell Diaz Leines:** Software (equal); writing – review and editing (equal). **Joseph Ellaway:** Software (equal); visualization (equal). **Genevieve Evans:** Data curation (equal). **Romana Gaborova:** Data curation (equal); formal analysis (equal). **Deepti Gupta:** Data curation (equal); writing – review and editing (supporting). **Aleksandras Gutmanas:** Project administration (supporting); supervision (supporting). **Deborah Harrus:** Data curation (equal); project administration (supporting). **Gerard J. Kleywegt:** Writing – review and editing (equal). **Weslley Morellato Bueno:** Software (equal). **Nurul Nadzirin:** Software (equal). **Sreenath S Nair:** Software (lead); visualization (equal). **Lukas Pravda:** Formal analysis (equal); software (equal). **Marcelo Querino Lima Afonso:** Software (equal). **David Sehnal:** Software (equal); visualization (equal). **Ahsan Tanweer:** Software (equal). **James Tolchard:** Data curation (equal); software (supporting). **Charlotte Abrams:** Writing – review and editing (supporting). **Roisin Dunlop:** Writing – review and editing (supporting). **Sameer Velankar:** Conceptualization (equal); funding acquisition (lead); investigation (equal); resources (lead); software (equal); supervision (equal).

## FUNDING INFORMATION

This work was supported by funding from the European Molecular Biology Laboratory‐European Bioinformatics Institute, by the Wellcome Trust via grants PDBe‐3D [104948/Z/14/Z], PDBe [218303/Z/19/Z], PDBe‐KB [223739/Z/21/Z], and FunSites [221327/Z/20/Z]; by the Biotechnology and Biological Sciences Research Council (BBSRC) through grants SIFTS [BB/M011674/1], PDBe‐India [BB/P025846/1], FunPDBe [BB/P024351/1], CATH‐BBR [BB/R015201/1], Structure Coverage [BB/S017135/1], 3D‐Gateway [BB/S020071/1], BioChemGRAPH [BB/T01959X/1], PDB‐FACT [BB/V004247/1], FunCLAN [BB/V016113/1], 3D‐Proteomics [BB/V018779/1], and PDBe‐RCSB [BB/W017970/1].

## Supporting information


Supplementary material S1

Supplementary material S2

## Data Availability

All of the data provided is freely available for both academic and commercial use under Creative Commons Attribution 4.0 (CC‐BY 4.0) license terms.
