# Supplementary material for: PDBe and PDBe‐KB: Providing high‐quality, up‐to‐date and integrated resources of macromolecular structures to support basic and applied research and education
Source: Protein Sci. 2022 Sep 28;31(10):e4439. doi: 10.1002/pro.4439 (PMC9517934; doi:10.1002/pro.4439)
Supplement: Supplementary file 1 — Supplementary material S1 [file PRO-31-e4439-s001.docx]

Supplementary material 1

Cypher query to find five examples of ligand pairs where the ligands have the same Murcko scaffold and are interacting with the same UniProt sequence residue, observed in different PDB entries.

MATCH (c1:ChemicalComponent)<-[:BOUNDED_BY]-(u:UNPResidue)<-[:MAP_TO_UNIPROT_RESIDUE]-(p1:PDBResidue)<-[:HAS_ARP_CONTACT]-(b1:BoundLigand)<-[:IS_AN_INSTANCE_OF]-(e1:Entity)

WITH c1, u, p1, b1, e1

MATCH (c1)-[:HAS_SIMILARITY]-(c2:ChemicalComponent)<-[:BOUNDED_BY]-(u)

RETURN *

LIMIT 5
