# Supplementary material for: PDBe and PDBe‐KB: Providing high‐quality, up‐to‐date and integrated resources of macromolecular structures to support basic and applied research and education
Source: Protein Sci. 2022 Sep 28;31(10):e4439. doi: 10.1002/pro.4439 (PMC9517934; doi:10.1002/pro.4439)
Supplement: Supplementary file 2 — Supplementary material S2 [file PRO-31-e4439-s002.docx]

Supplementary material 1


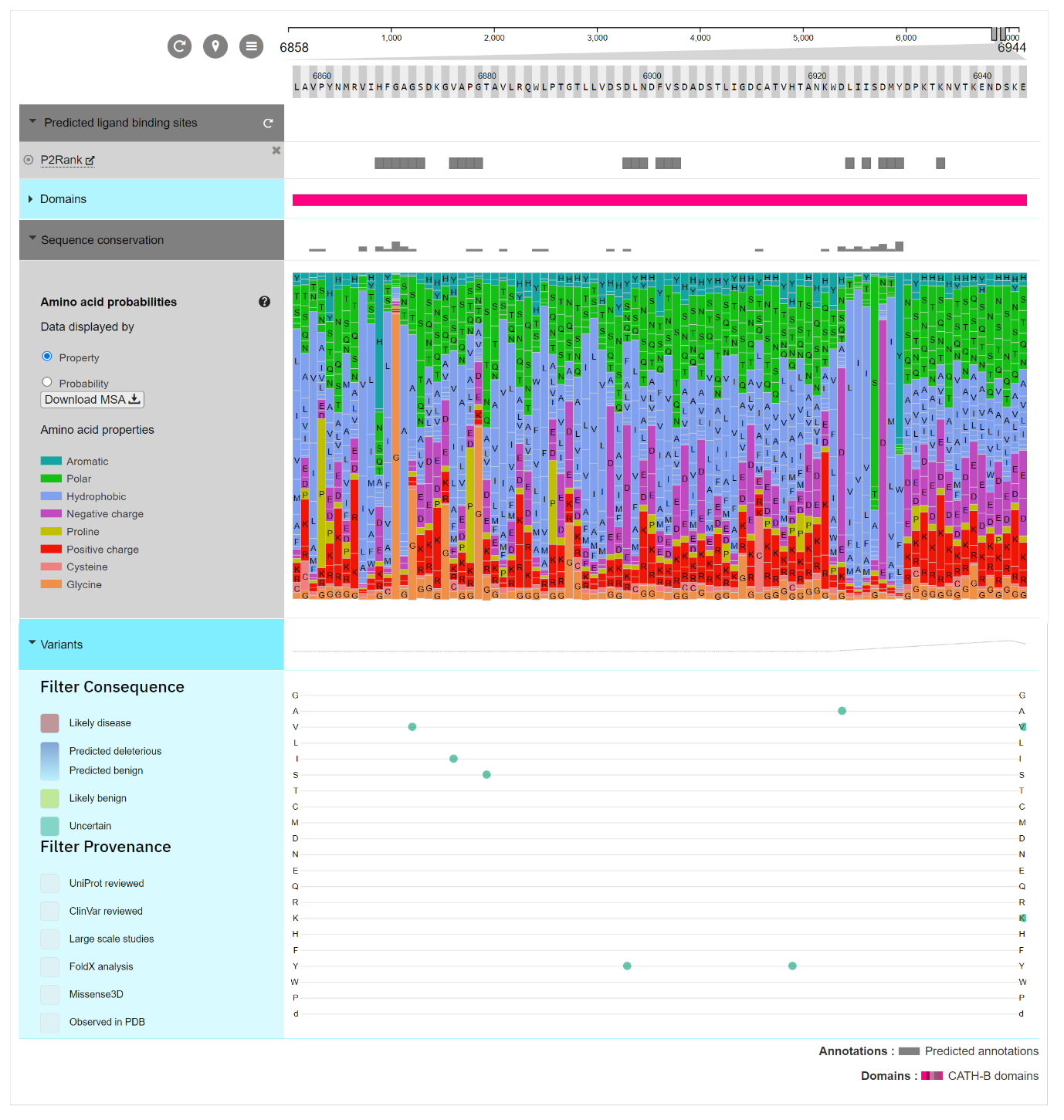


**Supplementary material 2 – PDB ProtVista data-visualisation tracks**

From top to bottom: 1.) site/residue-based track; 2.) segment-based track; 3.) sequence-conservation track; 4.) variants track.
